# Genome-wide association study of aggressive behaviour in chicken

**DOI:** 10.1038/srep30981

**Published:** 2016-08-03

**Authors:** Zhenhui Li, Ming Zheng, Bahareldin Ali Abdalla, Zhe Zhang, Zhenqiang Xu, Qiao Ye, Haiping Xu, Wei Luo, Qinghua Nie, Xiquan Zhang

**Affiliations:** 1 Department of Animal Genetics, Breeding and Reproduction, College of Animal Science, South China Agricultural University, Guangzhou 510642, Guangdong, China; 2 Guangdong Provincial Key Lab of Agro-Animal Genomics and Molecular Breeding and Key Lab of Chicken Genetics, Breeding and Reproduction, Ministry of Agriculture, Guangzhou 510642, Guangdong, China; 3 Wens NanFang Poultry Breeding Co., Ltd., YunFu 527400, Guangdong, China

## Abstract

In the poultry industry, aggressive behaviour is a large animal welfare issue all over the world. To date, little is known about the underlying genetics of the aggressive behaviour. Here, we performed a genome-wide association study (GWAS) to explore the genetic mechanism associated with aggressive behaviour in chickens. The GWAS results showed that a total of 33 SNPs were associated with aggressive behaviour traits (*P* < 4.6E-6). rs312463697 on chromosome 4 was significantly associated with aggression (*P* = 2.10905E-07), and it was in the intron region of the sortilin-related VPS10 domain containing receptor 2 (*SORCS2)* gene. In addition, biological function analysis of the nearest 26 genes around the significant SNPs was performed with Ingenuity Pathway Analysis. An interaction network contained 17 genes was obtained and *SORCS2* was involved in this network, interacted with nerve growth factor (*NGF*), nerve growth factor receptor (*NGFR*), dopa decarboxylase *(L-dopa*) and dopamine. After knockdown of *SORCS2*, the mRNA levels of *NGF, L-dopa* and dopamine receptor genes *DRD1, DRD2, DRD3 and DRD4* were significantly decreased (*P* < 0.05). In summary, our data indicated that *SORCS2* might play an important role in chicken aggressive behaviour through the regulation of dopaminergic pathways and *NGF*.

Aggressive behaviour is a large animal welfare issue affecting the poultry breeding population all over the world. Broiler breeder males under commercial conditions are reported to behave high levels of aggression, often injuring and sometimes killing females and also reduced fertility in a flock^1^,^2^.

Aggression is an evolutionarily conserved behaviour and it has been previously studied in many non-human species such as rodents, songbirds, zebrafish, and drosophila^3^. Chicken aggressive behaviour is defined as fight for living space, direct social dominance, food, strangeness, copulations, presence of male and other factors for individual survival condition^1^,^4^. It is an important component of chicken social behaviour through fighting with their companions to establish their position in the hierarchy and setting up social rank^5^. However, it could cause increased social stress, body damage, mortality as well as appearance defects, resulting in serious economic losses. Therefore, genetic mechanism regulating aggression can not only develop a better understanding of chicken aggression, but also can improves the economic efficiency and animal welfare for poultry industry.

It’s believed that, the problems associated with aggressiveness in broiler breeder males are genetic factors that produce differences in both general aggressiveness and sexual aggressiveness^6^. However, both genetic and environmental factors, including diet^7^, lighting conditions^8^, feeding methods, group size^9^, sex-mixed in a large flock of laying hens^10^, gender, and age, could modulate chicken aggressive behaviour. In chicken, an aggression-related heritability estimate value of 0.57 has been reported earlier by Siegel; in which selection trial of two-way was used to produce the next generation, and the selection is made with respect to only one parent for the selected trait from twice the ratio of change per generation to the average differential of selection^11^. Previous studies demonstrated that fear-related aggressive behaviour and early life stress-induced aggression were regulated by hypothalamic-pituitary-adrenal (HPA) axis^12^,^13^. Canonical neurotransmitters, such as dopamine, serotonin (5-HT) and gamma-aminobutyric acid (GABA), could modulate animal aggressive behaviour^14^. GWAS that screening majority of the genome using dense genomic markers have been developed and utilized widely in the analyses of complex traits in both animals and humans^15^. GWAS take vantage of a large numbers of SNP markers in population-wide linkage disequilibrium with extremely narrow regions potentially harboring candidate loci for the complex traits. To our knowledge, this is the first GWAS conducted to explore genetics and molecular mechanisms that associated with aggressive behaviour in chickens and we hope that our study can provides a new insight into understanding aggression in chickens.

In the present study, with the use of a 600 K Affymetrix^®^ Axiom^®^ High density (HD) chicken genotyping array, we performed GWAS to identify candidate genes or genomic regions that associated with chicken aggressive behaviour. One SNP rs312463697 was found to be reached 5% Bonferroni genome-wide significantly associated (*P* = 2.10905E-07) with male aggression and it is located in the intron region of the *SORCS2* gene on chromosome 4. In response to knockdown of *SORCS2* by siRNA, the mRNA levels of *NGF, L-dopa* and dopamine receptor genes (*DRD1, DRD2, DRD3 and DRD4*) were significantly decreased (*P* < 0.05). In summary, our data indicated that variations of *SORCS2* gene might contribute to the susceptibility of chicken aggressive behaviour and these can provide a new insight into genetics of aggressive behaviour in chickens.

## Results

To explore the genetic regulatory mechanism associated with aggressive behaviour, a total of 265 male chickens were genotyped with a 600 K Affymetrix^®^ Axiom^®^ HD chicken genotyping array consisting of 559,898 loci. After filtering, 468,020 SNPs were used for further analysis. Behavioural observations and growth traits of male chickens were recorded daily from the adult males for 16 days. The parameters of male aggressive behaviour measured traits were used for GWAS-association study (Table 1). *SORCS2* knockdown was tested in this study. The gene networks and gene expression of some of the top candidates related to aggressive behaviour have also been investigated. Here, 33 SNPs were significantly associated with male’s aggressive behaviour. Biological function analysis of the nearest (26 genes) genes of significant SNPs was performed with IPA. An interaction network contained 17 genes was obtained and *SORCS2* was involved in this network, and interacted with *NGF, NGFR, L-dopa* and *dopamine*. Further, we also measured *SORCS2* mRNA levels using RT-qPCR method, results showed that the highest aggressive chickens have significantly higher expression level of *SORCS2* in the pituitary tissue than the lowest aggressive chickens (*P* = 0.029). Moreover, after knockdown of *SORCS2*, the mRNA levels of *NGF*, *L-dopa* and dopamine receptor genes (*DRD1, DRD2, DRD3 and DRD4*) were significantly decreased (*P* = 0.003, 0.023 and 0.012, 1.64981E-05, 0.045, and 6.67515E-05), respectively.

### Correlation analysis

In order to illuminate the correlation between chicken aggressive behaviour and growth traits, chicken daily aggressive frequency (DAF), daily feed intake (DFI), daily body weight (DBW) and daily body weight gain (DBWG) were measured and analyzed by Pearson correlation test. Analysis of Pearson’s correlation coefficient showed that there was significant positive correlation (*P* = 0.0105) between DAF and DFI. Interestingly, there is no significant correlation between DAF and DBW (*P* = 0.9785), or between DAF and DBWG (*P* = 0.6111) (Table 2). These data indicated that aggressive chicken consumed more feeds, but did not converted into muscle tissue or body weight gain.

### Genome-wide association analysis (GWAS)

**Figure 1:**
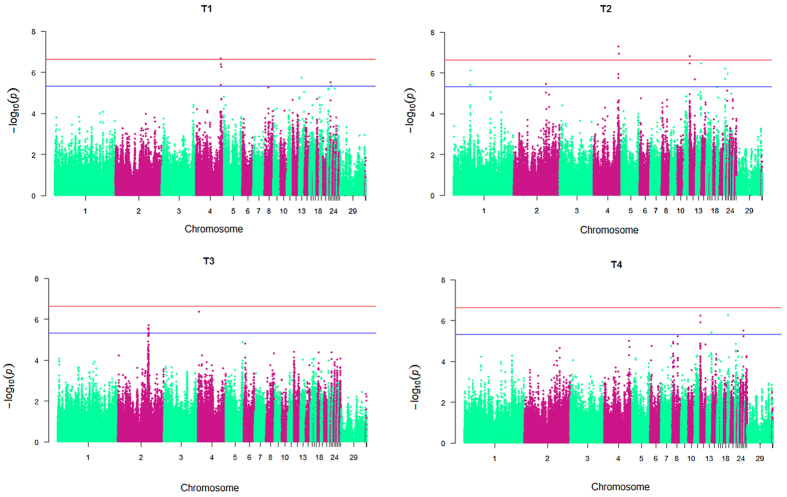
Manhattan plots of genome-wide association study on chicken aggressive-behaviour measured traits from T1 to T4 for all the SNPs. The associated values (in terms of −log10P) are shown by chromosomes. The blue highlighted line indicates genome-wide association (*P* = 4.6E-6), and the red highlighted line indicates significance with a *P*-value threshold of the 5% Bonferroni genome-wide significance (*P* = 2.3E-7).

The 265 male Chinese native dwarf yellow chickens were genotyped using a 600 K Affymetrix^®^ Axiom^®^ HD chicken genotyping array. Genotyping revealed a set of 559,898 scorable SNPs. After quality control filtering, 468,020 SNPs were finally used for GWAS to study aggressive behaviour trait. All birds were healthy, with no suffer from illness. Chromosomal position for each SNP marker was obtained from the chicken reference genome (ftp://ftp.ensembl.org/pub/release-73/fasta/gallus_gallus/dna/). 552,395 SNP markers were mapped to known chromosomal positions, while remaining 7,503 SNPs were mapped to unknown (UN) chromosomal positions. The average physical distance between adjacent SNPs was 2.21 Kb. The number of SNP markers per chromosome with known positions ranged from 14 on chromosome W to 98,565 on chromosome 1, and the average spacing for each SNP marker was 2.23 Kb (Supplementary Table S1). There was no dramatic deviation between observed and expected (−log_10_ *P*-value) in the quantile-quantile plot and the estimate of λ of each trait is 1.00000 (Fig. S1), suggesting that there was little or no evidence of residual population structure effects in test statistic inflation^16^. The global view of *P*-values for all SNP markers of four aggressive-behaviour phenotypes measured traits were visualized by a Manhattan plot (Fig. 1) using the “qqman” package in R Language^17^, and the results showed that the chicken (*Gallus Gallus)* chromosome 4 (GG4) region was the most frequent associated with aggressive behaviour, followed by chromosome 2 (GGA2) and chromosome 12 (GGA12) (Table 3). A total of 40 SNP effects, involving 33 SNPs and 26 genes were detected for the four aggressive-behaviour phenotypes measured traits with genome-wide significance (*P* < 4.6E-6) (Table 3). Among the 33 suggestive significant associated SNPs, 65%, 32.5%, and 2.5% were transition, transversion and insertion, respectively (Table 3). Two SNP effects involving one SNP located in the *SORCS2* reached genome-wide significance for T1 and T2 of aggressive-behaviour phenotypes measured traits. The top five significant SNPs were found in the regions of known genes; SNP_1_ found in the intron region of the carboxypeptidase Z gene (*CPZ*) (*P* = 5.15392E-08), SNP_2_ found at 17,602 bp upstream of G protein-coupled receptor 78 (*GPR78*) (*P* = 5.15392E-08). SNP_3_ is a synonymous SNP of huntingtin (*HTT*) (*P* = 1.12185E-07), SNP_4_ is a synonymous SNP of signal peptidase complex subunit 1 (*SPCS1*) (*P* = 1.55329E-07) and SNP_5_ at the intron region of *SORCS2* (*P* = 2.10905E-07).

The distribution of genome-wide significant SNPs spread across 10 chromosomes, including chromosome 1, 2, 4, 12, 13, 19, 21–23 and 26. The GWAS results revealed that the largest cluster of significant SNP effects for chicken aggression traits involved 10 SNPs in the region of 3,773,061–81,759,949 bp on GGA4, including 9 genes (Table 3).

### Ingenuity Pathway Analysis (IPA)

**Figure 2:**
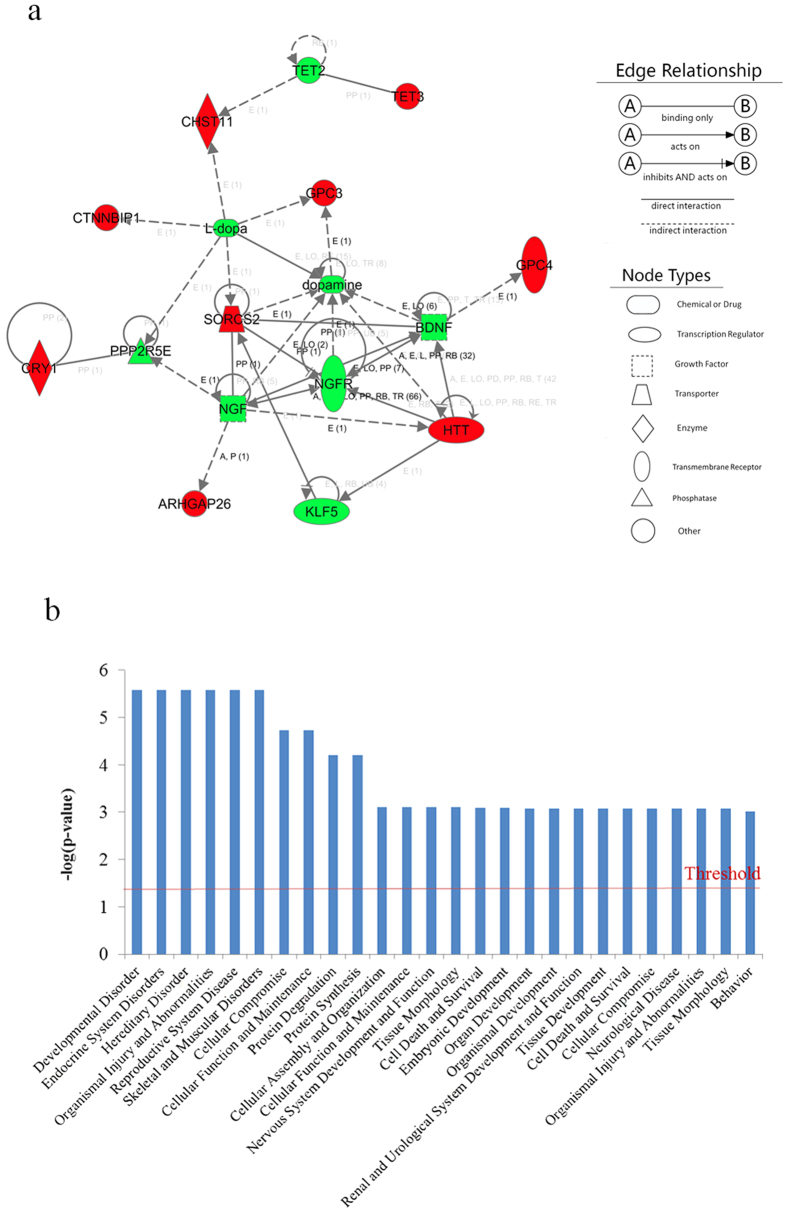
Gene-gene interaction network analysis. (**a)** The gene-gene interaction network identified by Ingenuity Pathway Analysis (IPA) online software. The input was the genes nearest to the SNPs that reached suggestive genome-wide significance. Imported each gene identifier was mapped to its corresponding gene object in Ingenuity Pathways Knowledge Base (IPKB) and overlaid onto global molecular network developed from information contained in the IPKB. The red color shaded notes are the genes nearest to GWAS SNPs and also involved in IPA interaction network. The green color shaded notes are not from the genes nearest to GWAS SNPs but are transcription factors that are associated with the regulation of some of these genes identified by IPA algorithm. Edge type and node type descriptions illustrate the nature of the relationship between genes and their functions. Solid lines indicated direct interaction, while dashed lines indicated indirect interaction. **(b)** The top 26 represented functional groups of the genes nearest to GWAS SNPs were generated by IPA.

The genome-wide association mapping revealed that 40 SNP effects, involving 33 SNPs and 26 genes, were significantly associated with chicken aggressive behaviours measured traits. To identify the function of the nearest genes to the 33 associated loci and their potential connections, we used IPA to analyze the 26 genes within the significance associated loci. In this study, there was a gene-gene interaction network with score of 28 generated by IPA (Fig. 2a). The gene-gene interaction network contained 9 of the nearest genes including *SORCS2, HTT, tet methylcytosine dioxygenase 3 (TET3), carbohydrate (chondroitin 4) sulfotransferase 11 (CHST11), catenin, beta interacting protein 1 (CTNNBIP1), glypican 3 (GPC3), glypican 4 (GPC4), cryptochrome circadian clock1 (CRY1) and Rho GTPase activating protein 26 (ARHGAP26)* (Fig. 2a) (Table 4). In the network, *SORCS2* interacted with *NGF, NGFR, L-dopa* and *dopamine* (Fig. 2a). A total of 80 functions/diseases were significantly represented in the network eligible genes, and the 26 top of them were shown in Fig. 2b. Importantly, the function of “behaviour” was the 26^th^ top functions/diseases represented in the gene-gene interaction network (Fig. 2b). These results indicated that *SORCS2* could affect the expression of *NGF*, *NGFR, L-dopa* and *dopamine*, and might further play an important role in the regulation of chicken aggressive behavior.

### Knockdown of *SORCS2* inhibited the expression of *NGF, L-dopa* and dopamine receptor family

**Figure 3:**
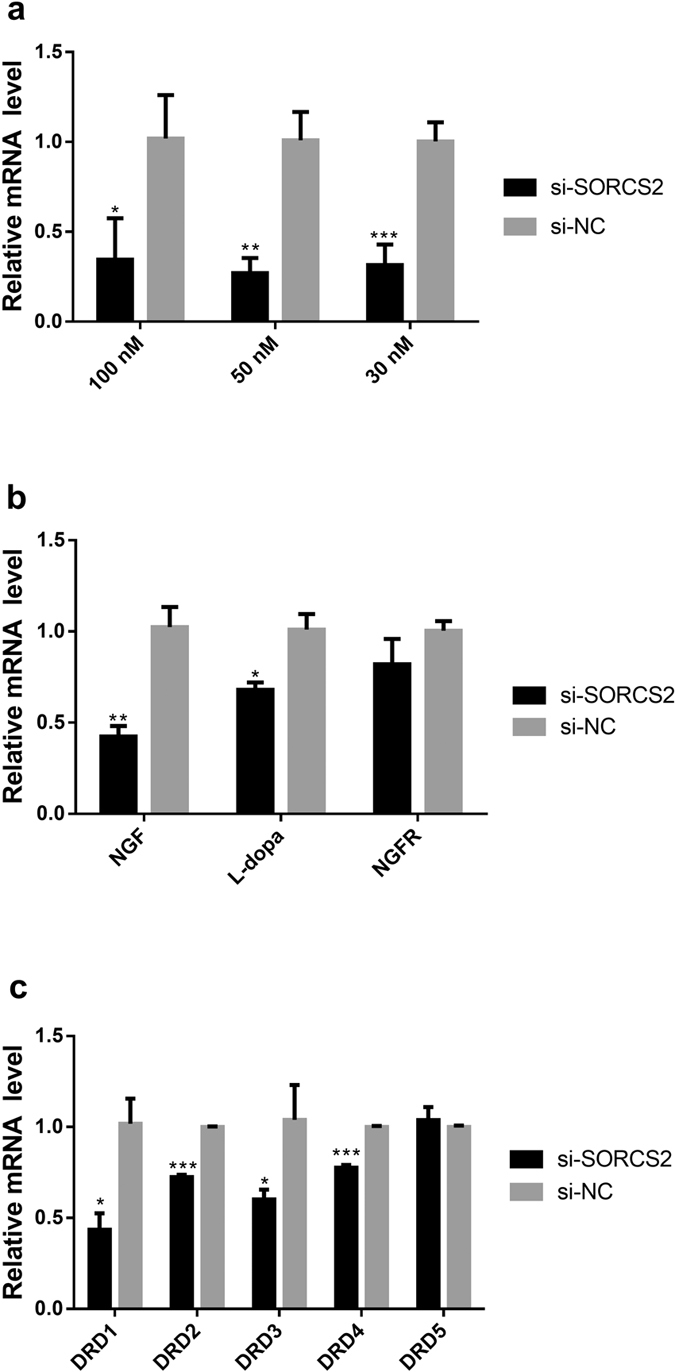
Expression levels of chicken *SORCS2, NGF, NGFR, L-dopa* and dopamine receptor family in *SORCS2* knockdown DF-1 cells. (**a)** In total, 100 nM, 50 nM, 30 nM of si-SORCS2 could significantly decrease *SORCS2* expression levels in DF-1 cells at 48 h after transfection. **(b)** Knockdown of *SORCS2* decreases the expression level of *NGF, NGFR and L-dapa.* (**c**) Knockdown of SORCS2 decreases the expression level of dopamine receptor family (*DRD1, DRD2, DRD3 and DRD4*). The levels of mRNA were measured by RT-qPCR analysis. In all panels, the levels of mRNA were measured by RT-qPCR analysis. Quantitative expressions of the data are presented as means ± S.E.M of at least three biological replicates. *, **, and *** indicate *P*-value significance at the threshold levels of 0.05, 0.01 and 0.001, respectively.

In this study, *SORCS2* is involved in the IPA network and interacted with *NGF, NGFR, L-dopa and dopamine*. Previous study reported that the *NGF* mRNA increased in the hypothalamus of mouse used as model for studying aggression^18^. To investigate the expression correlation between *SORCS2* and *NGF* or other candidate genes in the gene-gene interaction network significantly associated with male aggression, for this purpose, we knocked down *SORCS2* by si-SORCS2 oligoribonucleotides in DF-1 cells and then explored the mRNA expression alteration of *NGF, NGFR, L-dopa* and dopamine receptor family. The transfection efficiency analysis of si-SORCS2 showed that concentrations of 100 nM, 50 nM and 30 nM were significantly decreases SORCS2 mRNA expression levels in DF-1 cells at 48 h after transfection (*P* = 0.0124, 0.0010, 0.0008), respectively (Fig. 3a). On the other hand, in response to *SORCS2* knockdown (30 nM), the mRNA levels of *NGF and L-dopa* (Fig. 3b) significantly decreased (*P* = 0.003, and 0.023), and with same effect to the dopamine receptor genes (*DRD1, DRD2, DRD3 and DRD4)* (Fig. 3c) (*P*  = 0.012, 1.64981E-05, 0.045 and 6.67515E-05), however, the expression of *NGFR* was down-regulated by si-SORCS2, but statistically was not-significant (*P* = 0.184). These results suggested that *SORCS2* might affect the expression of *NGF, L-dopa* and dopamine receptor genes and then affect the regulation of chicken aggression.

### *SORCS2* is up-regulated in aggressive chicken

**Figure 4:**
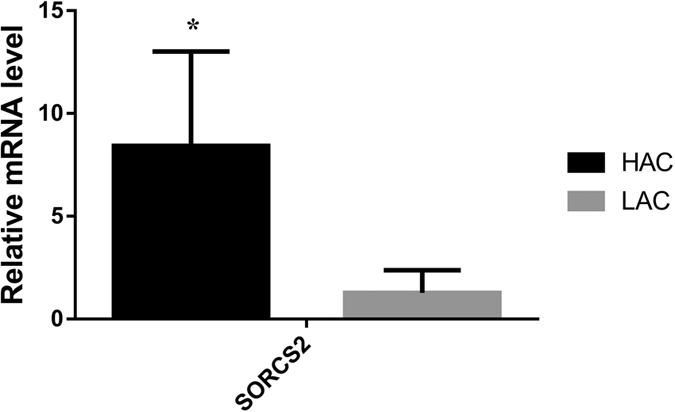
*SORCS2* is up-regulated in aggressive chickens. The expression level of *SORCS2* is up-regulated in highest aggressive chickens (HAC) when compared with lowest aggressive chickens (LAC). The levels of mRNA were measured by RT-qPCR analysis. Quantitative expressions of the data are presented as means ± S.E.M of at least three biological replicates. ^*^P < 0.05 was deemed to be significant.

The above result shows two SNP effects involving *SORCS2* were significantly associated with chicken aggressive behaviour, and *SORCS2* could affect the expression of *NGF, L-dopa and* dopamine receptor genes. However, we tested the mRNA expression difference of *SORCS2* between the highest aggressive chicken (HAC) and the lowest aggressive chicken (LAC). Our qPCR results showed that the highest aggressive chicken has significantly higher expression level of *SORCS2* in the pituitary tissue than the lowest aggressive chicken (*P* = 0.029) are presented in Fig. 4.

## Discussion

This study provides new information on genetics of aggressive behaviour in chickens. After performing GWAS, we found that one SNP located at rs312463697 in the intron region of the *SORCS2* gene on chromosome 4, significantly (*P* = 2.1091E-07) associated with aggressive behaviour traits in males. An interaction network contained 17 genes was obtained and *SORCS2* was involved in this network, and interacted with *NGF, NGFR, L-dopa* and *dopamine* genes. After knockdown of *SORCS2*, the mRNA levels of *NGF, L-dopa* and dopamine receptor genes were dramatically decreased. Furthermore, we found that an excessive aggression could cause low feed conversion rate. The automatic feeding recording system used in this study led to an increase density of birds, resulting in increased aggressive behaviour; previous study in chicken confirming a similar observation, which they studied the effect of inter-individual distances in laying-hens housed in groups of three in pens of two different sizes, result shows birds placed in small size pen tended to display some force, or combination of forces higher than that of the large size pen^19^. Food is one of the most considerable factors of aggressive behaviour^20^. In the present study, a significant positive correlation was found between DAF and DFI, but not between DAF and DBW, or between DAF and DBWG. This may be due to that aggressive chicken required more energy for fighting, and they have to increase feed intake in an effort to meet their energy requirements. Poultry feeds usually account for 70% of the total production cost, and chicken aggressive behaviour could increase feed intake and leads to greater waste of money. Our data revealed that aggressive behaviour could increase feed intake and in turn, reduce the economic benefit in poultry production. Therefore, characterization of genes and/or identifying genomic regions controlling aggressive behaviour and further reduce chicken aggression has an important economic impact on poultry production.

Since no GWAS study for aggressive behaviour in chickens has been previously published, we have made an attempt to compare our results specifically GWAS candidate genes with those of previously reported genes or genomic related information. GWAS is a powerful technique that allows us to discover hundreds of common variants whose allele frequencies are statistically correlated with various illnesses and traits. Chicken aggression is a common phenomenon in poultry production, and it can lead to body damage as well as appearance defects, resulting in serious economic losses. In the present study, GWAS was performed to investigate variations may coexist within a population associated with chicken aggressive behaviour. Consequently, a total of 40 SNP effects significantly associated with aggressive behaviour were identified.

*SORCS2* is a type I trans-membrane glycoprotein receptor that belongs to the mammalian Vps10p family^21^, they plays a key role in brain disease, such as attention-deficit hyperactivity disorder (ADHD), bipolar disorder (BPD), schizophrenia and Alzheimer disease (AD)^22^,^23^,^24^,^25^. SORCS2 protein was firstly isolated from murine brain^26^ and highly expressed in the developing and mature murine central nervous system^21^. The expression of *SORCS2* has also been found in the kidney, lung, testis and heart, with lower level than brain^27^. In the present study, we firstly found that the highest aggressive chicken has significantly higher expression level of *SORCS2* in the pituitary than the lowest aggressive chicken. In human, *SORCS2* protein is predominantly expressed in the developing and mature murine central nervous system^21^, while it was also expressed in kidney, lung, testis and heart tissues^27^.

In this study, the expression of four dopamine receptors, *DRD1, DRD2, DRD3* and *DRD4*, were also found to be reduced after *SORCS2* knockdown. Intriguingly, there is no indeed expression difference of *DRD5* after *SORCS2* knockdown in DF-1 cells. Dopamine is an organic chemical of the catecholamine and phenethylamine families that plays several essential roles in the brain and body, it is an amine synthesized by eliminating a carboxyl group from a molecule of its precursor chemical L-dopa, which is synthesized in the brain and kidneys. In addition, it was demonstrated to be linked with aggression in various species^28^. Dopamine exerts its action by binding to specific membrane receptors, the dopamine receptor family. In addition, the dopaminergic and opioidergic reward pathways, which controlled the fundamental living ability, such as eating, loving, reproduction, were critical for survival. However, some risk taking behaviours, such as alcohol, drugs and gambling, could damage dopaminergic and opioidergic reward pathways^29^. Five distinct dopamine receptors (*DRD1, DRD2, DRD3, DRD4*, and *DRD5*) have been isolated and all of them are seven transmembrane domain (7TM) G-protein coupled receptors. Previous study reported that *SORCS2* regulates dopaminergic wiring in peripheral glia^30^. Importantly, variation in *DRD4* has been proved to be associated with chicken feather pecking behavioural disorder^31^. However, the correlation between *SORCS2* and dopamine receptors in chicken aggression regulation remains unclear. On the other hand, numerous animal studies indicated that dopaminergic neurotransmission was involved in the regulation of impulsive aggression and violence^32^. Therefore, dopaminergic gene expression variation can modify many complex traits including impulsivity and aggression. As far as we know, dopamine receptor D1, D2, D3 and D4 have been proved to be associated with aggressive in many species^33^,^34^,^35^,^36^. To our knowledge, the association between *DRD5* and aggression trait has not been reported. Previous study revealed that *SORCS2* plays an important role in dopaminergic wiring expression, and its knockout in mice caused reduction in dopamine levels^30^. These data suggested that *SORCS2* was important for the maintenance of dopamine receptors including *DRD1, DRD2, DRD3* and *DRD4*, and that change of *SORCS2* expression might induce profound changes in dopaminergic pathways.

In IPA analysis, 9 genes, which were the nearest genes to significant SNPs, involved in the gene-gene interaction network. It is noteworthy that *SORCS2* directly interacts with *NGF*, *NGFR, L-dopa and dopamine* in the gene-gene interaction network. In response to *SORCS2* knockdown, the mRNA expressions of *NGF, L-dopa and* dopamine receptor family were significantly reduced (*P* < 0.05), while the expression of *NGFR* was down-regulated, although this down-regulation was not significant (Fig. 3b). *NGF* is a member of the neurotrophin (NT) family secreted by a neuron’s target cell, promotes neuronal survival, differentiation, axon guidance and synaptic strengthening by binding to its two neurotrophin receptors: (NGFR) p75^NTR^ or p140^Trk^ (TrkA)^37^. Previous studies found that stress could produce significant alterations in circulating *NGF* levels^38^, and changes of *NGF* expression level in mouse hypothalamus could induce intermale aggressive behaviour^39^. On the other hand, *SORCS2* was demonstrated to act as a proneurotrophin (proNT) receptor to regulate both tropic and apoptotic signals in cooperation with *NGF* receptor^30^.

Our *SORCS2* knockdown result showed that *L-dopa* expression level was decreased. Previous studies have reported that various doses of *L-dopa* suppress aggressive behaviour in mice^40^,^41^. *L-dopa* is a chemical that is made and used as part of the normal biology of humans, some animals and plants. *L-dopa* is the precursor to dopamine, norepinephrine, and epinephrine collectively known as catecholamines. *L-dopa* mediates neurotrophic factor release by the brain and CNS. Surprisingly, the effects of our all GWAS-IPA-candidate genes (*SORSC2, NGF, NGFR, dopamine and L-dopa*) on aggressive behaviour in chicken had not been investigated before.

In conclusion, we found that a significant positive correlation between daily aggressive frequency and daily feed intake (*P* < 0.05), but not between daily aggressive frequency and daily body weight gain. A total of 40 SNP effects were demonstrated to be significantly associated with chicken aggressive behaviour (*P* < 4.6E-6). One SNP was found to be located at rs312463697 in the intron region of *SORCS2* on chromosome 4, and it is 5% Bonferroni genome-wide significantly associated with male aggression (*P* = 2.10905E-07). Furthermore, the highest aggressive chicken has significantly higher expression level of *SORCS2* in the pituitary than the lowest aggressive chicken (*P* < 0.05). The interaction between *SORCS2* and *NGF, NGFR, L-dopa and dopamine* were obtained by IPA and the expressions of *NGF, L-dopa* and dopamine receptor family (*DRD1, DRD2, DRD3*, *DRD4)* were significantly reduced after *SORCS2* knockdown, indicating that *SORCS2* might play an important role in chicken aggressive behaviour through the regulation of dopaminergic pathways and *NGF*. These data can provide a new insight into molecular mechanisms of chicken aggression.

## Methods

### Ethics statement

The experimental procedures used in this study met the guidelines of the Animal Care and Use Committee of the South China Agricultural University (SCAU) (Guangzhou, People’s Republic of China). All animal experiments of this study were approved by the Animal Care and Use Committee of the SCAU with approval number SCAU#0017. All efforts were made to minimize animal suffering.

### Experimental animals

A total of 300 male chicks at one-day-old from the 25^th^ generation of Chinese native breed a dwarf yellow meat-type chicken (strain number, N301) were obtained from the Wens Nanfang Poultry Breeding Co. Ltd (Yun Fu city, Guangdong province, China). All chicks were reared in 4 pens (3.9 m × 2.3 m) covered with a 7 cm layer of 100% natural pine wood shavings until 42 days of age, the begin of the behavioural testing. At 42 days of age, 265 male chickens were selected and transferred to semi-enclosed broiler house (17 m × 3 m), fore close association between birds which promotes aggressive behaviour to be displayed^4^,^11^. Automatic feeding recording system was used to identify individuals and recording daily body weight and daily feed intake by an infrared (detector) scanning electronic chip (Guangxing Poultry Equipment Group CO., LTD; Guangdong Province, China), which was inserted between two wattles of each male (Supplementary Fig. S2). All birds were kept in an identical and constant local environment and fed *ad libitum* a commercial diet consisting of three phases until 83 days of age, the end of the experimental period. Clean water (fresh) was available *ad libitum* to the birds.

### Aggressive behaviour trait

Behavioural testing of 265 male chickens was begun when the birds were at 60 days of age, in order to record the aggressive behaviour for individuals; each male was feet-banded with specific color (Supplementary Fig. S3a) as previously described with some modification^42^. All birds were given 7 days to adapt to the new condition. After the adaptation period, male aggression behaviour was recorded from 67 to 82 days of age, by a team of skilled observers (3 persons) and each of them was responsible for recording the aggression trait after standing on the middle of the one third area (5.7 m × 3.0 m) of the chickens floor pen (Supplementary Fig. S3b). Before the male aggressive recording process, the observers waited for 5 to 8 minutes until the chicken’s activity was unaffected by the existence of the observers. The definition of aggressive behaviour is based on the ethogram of Väisänen^43^, where no feather pulling was involved. The following behavioural features were recorded in this study: including threats, attacks, chase, aggressive peck, fight and leap. So, the aggressive behaviours were observed two times a day for 16 days; testing took place between 9:00 and 11:00 am, and 03:00 and 05:00 pm. In order to efficiently explore the aggressive behaviour-related potential loci, the behavioural measurements were divided into four aggressive-behaviour phenotypes measured traits (T) and used for GWAS, including T1 = number of fighting times during the whole recording period (16 days), T2 = number of fighting times in days with frequencies not less than 4 times per day, T3 = number of days for chicken showed fighting. In T4 = number of days for chicken showed fighting with frequencies not less than 4 times per day (Table 1). At 83 days of age, one mL blood samples were withdrawn via wing veins of all male chickens (265 birds) into syringes containing 1.5% EDTA used as an anticoagulant. In addition, according to the aggression record, pituitary samples of 3 highest aggressive chickens (HAC) and 3 lowest aggressive chickens (LAC) were collected and stored at −80 °C until RNA extraction.

### Correlation analysis

We hypothesized that aggressive behaviour may have strong relation with chicken growth performance. In order to test this hypothesis, we analyzed the correlation between daily aggressive frequency (DAF) (data of T1 ~ T4) and growth performance of 265 male chickens; including daily feed intake (DFI), daily body weight (DBW) and daily body weight gain (DBWG). The Pearson correlation test was performed using cor.test function in R language (http://www.r-project.org)^44^. The Pearson r calculated using the following formula:


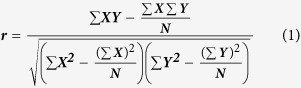


The Pearson correlation test calculates a coefficient value ranging from +1 to −1, and positive value means a positive correlation while negative value means an inverse correlation. Correlation analysis of *P* values was obtained using cor.test command in R language.

### Genotyping and quality control of social aggressiveness in male chickens

Genomic DNA was extracted from the above collected blood samples using the NRBC Blood DNA Kit (Omega Bio-Tek, Norcross, GA, USA) according to the manufacturer’s instructions. DNA samples were quantified for DNA concentrations and genotyped using a 600K Affymetrix® Axiom® HD chicken genotyping array, 559,898 SNPs were generated. SNPs were distributed on GGA1-28, chromosome Z, W, and two linkage group: LGE22 and LGE64. Genotyping of the SNPs was carried out by Shanghai Biotechnology Corporation (Shanghai, China). Quality control was assessed by GenABEL package of R software^45^, SNP selection required less than 5% minor allele frequency (MAF), 95% or more genotype call rate, and Hardy-Weinberg equilibrium (*P* > 0.00001). After quality control, 468,020 SNPs were finally selected for GWAS (Supplementary Table S1).

### Statistical and bioinformatic analysis

The statistical comparison of mean values such as mean mRNA expression levels among different genes was compared with one-way analysis of variance (ANOVA) ) in SAS statistics software (ver. 8.0, SAS Institute, Cary, NC, USA). Quantitative expressions of the data are presented as means ± S.E.M of at least three biological replicates. Values were considered significantly different with *P* values of less than 0.05., unless otherwise indicated.

The threshold *P*-value of the 5% Bonferroni genome-wide significance was calculated based on the “LD adjusted” Bonferroni method. Using this method, the total number of block and interblock SNP is 217,788 (Supplementary Table S2), so that the threshold *P*-values of 5% Bonferroni LD-adjusted genome-wide significant was 2.30E-7 (0.05/217,788). The threshold of *P*-value for the significance of “suggestive association” that allows one time false positive effect in GWAS test was calculated based on the same method as noted above and it was 4.60E-6 (1/217,788)^46^. These procedures were evaluated using PLINK^47^. Generalized linear mixed models (GLMMs) were used to detect SNP that correlated with chicken behavioural variables^48^. The model equation is expressed as following:


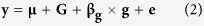


where **y** = the vector of phenotypes after normal transformation; **μ** = the mean of the population; **G** = the vector of poly gene background effects; **β**_**g**_ = the vector of allelic additive genetic effect; **g** = the genotype value: 0, 1 or 2; **e** = a vector of residual effects. **G** and **e** are random effects fitting the multinomial normal distribution, **μ **~ N (0, 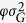) and **e **~ N (0, 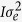). Association analysis was performed using GenABEL, a packages in the R software^45^.

The nearest genes of the suggestive significance association loci (GWAS SNPs) of male aggressive behaviour were used for gene interaction network and biological process enrichment analysis by Ingenuity Pathway Analysis (IPA) (www.ingenuity.com) each gene symbol was mapped to its corresponding gene object in IPA. More details about IPA see the supplementary information (supplementary IPA).

### RNA isolation, cDNA synthesis and quantitative real-time PCR

In order to investigate the relative mRNA expression levels of *SORCS2* between HAC and LAC, total RNA was extracted from frozen tissues of the pituitary samples using RNAiso reagent (TaKaRa, Osaka, Japan) according to the manufacture’s protocol. The obtained RNA quality was detected by 1.5% agarose gel electrophoresis and the concentration was determined by measuring the optical density in a NanoDrop 2000c spectrophotometer (Thermo Scientific™) at 260/280 nm ratio. One microgram of pooled RNA was used to synthesize cDNA by the PrimeScript™ RT reagent Kit with gDNA Eraser (Takara, Osaka, Japan) following the manufacture’s protocol. mRNA levels were analyzed with quantitative real-time PCR (qRT-PCR) and were performed on a CFX96 Real-Time Detection System (Bio-Rad) using KAPA SYBR® FAST Universal qPCR Kit (KAPABIOSYSTEMS, Boston, Massachusetts, United States) with *β*-actin used to normalize the relative abundance of mRNA in each reaction. The analysis of mRNA expression level was calculated using 2^−ΔΔCt^ method^49^. Primer sequences for RT-qPCR are shown in Supplementary Table S3.

### Cell culture and RNA oligoribonucleotides transfection

*The SORCS2, NGF, NGFR, L-dopa and dopamine genes* were predicted by IPA to have strongest correlations with male aggression. To confirm that we used the DF-1 cell line of chicken embryonic fibroblast for transfection study, the cell was obtained from the Bank of Committee on Type Culture Collection of the Chinese Academy of Sciences. Before transfection, DF-1 cells were seeded in 12-well plates and maintained in DMEM, supplemented with 10% fetal bovine serum (FBS), and 100 μg/mL penicillin/streptomycin (Invitrogen). All cells were incubated at 37 °C in a humidified atmosphere containing 5% CO_2_. siRNA target sequences are screened against the *SORCS2* gene (si-SORCS2) and siRNA non-specific control duplex (si-NC) were designed and synthesized by RiboBio (RiboBio, Guangzhou, China). When the DF-1 cells grew to 50% confluence, they were transfected with si-SORCS2 or si-NC using Lipofectamine 3000 reagent (Invitrogen) according to the manufacture’s protocol. 48 h after transfection, the *SORCS2* knockdown DF-1 cells were subjected to RNA isolation for RT-qPCR analysis. Differentiation markers *SORCS2* mRNA and si-NC mRNA were identified by RT-qPCR analysis and it are performed with similar procedures and analysis as mentioned above.

## Additional Information

**How to cite this article**: Li, Z. *et al*. Genome-wide association study of aggressive behaviour in chicken. *Sci. Rep.* **6**, 30981; doi: 10.1038/srep30981 (2016).

## Supplementary Material

Supplementary Information

## Figures and Tables

**Table 1:** Four aggressive-behaviour phenotypes measured traits in male chickens.

Abbreviation	Phenotype Description	Classification	Mean ± SD
T1	Number of fighting times during the whole recording period (16 days)	Fighting times	14.69 ± 11.24
T2	Number of fighting times in days with frequencies not less than 4 times per day		4.64 ± 8.63
T3	Number of days for chicken showed fighting	Fighting days	7.28 ± 3.53
T4	Number of days for chicken showed fighting with frequencies not less than 4 times per day		0.89 ± 3.53

**Table 2:** Pearson’s product-moment correlation between aggressive behaviour and growth traits, including daily feed intake (DFI), daily body weight (DBW) and daily body weight gain (DBWG).

Trait	FTD					
	r	p	t	df	95 percent confidence interval	
DFI	0.03930552	0.01048*	2.5608	4238	0.009214962	0.069324968
DBW	−0.000413292	0.9785	−0.026905	4238	−0.03051438	0.02968854
DBWG	−0.007812161	0.6111	−0.50859	4238	−0.03790471	0.02229455

“r” stands for the estimated measure of association; “t” stands for the value of the test statistic; “df” stands for the degrees of freedom of the test statistic in the case that it follows a t distribution; “p” stands for the *p*-value of the test; alternative hypothesis: true correlation is not equal to 0.

**Table 3:** SNPs with genome-wide significance effects of four aggressive-behaviour phenotypes measured traits in chickens.

Trait	CHR	Position (bp)	SNP ID	Allele	Location	The nearest gene to SNP	Distance (bp)	*P*-value
T1	4	79854217	Gga_rs312463697	C/T	Intronic	SORCS2	0	2.1091E-07
T1	4	80707803	Gga_rs317688790	G/T	Upstream	GPR78	17602	4.2035E-07
T1	4	80757827	Gga_rs16443048	A/G	Intronic	CPZ	0	4.2035E-07
T1	4	81759949	Gga_rs16444314	A/C	Synonymous codon	HTT	0	5.5543E-07
T1	13	9005033	Gga_rs313230353	A/G	Upstream	LOC101751961	12164	1.8815E-06
T1	22	2822519	Gga_rs16740250	G/T	Downstream	TET3	1151	3.0915E-06
T1	4	80665268	Gga_rs16442957	C/T	Downstream	LOC101750928	36839	4.1664E-06
T2	4	80707803	Gga_rs317688790	G/T	Upstream	GPR78	17602	5.1539E-08
T2	4	80757827	Gga_rs16443048	A/G	Intronic	CPZ	0	5.1539E-08
T2	4	81759949	Gga_rs16444314	A/C	Synonymous codon	HTT	0	1.1218E-07
T2	12	745686	Gga_rs313538765	C/T	Synonymous codon	SPCS1	0	1.5533E-07
T2	13	16756745	Gga_rs317892933	A/G	Intronic	ARHGAP26	0	3.5018E-07
T2	12	845905	Gga_rs317911278	C/G	Intronic	SFMBT1	0	3.5434E-07
T2	21	3548623	Gga_rs315850882	C/T	Intronic	CTNNBIP1	0	6.2698E-07
T2	21	3556743	Gga_rs318122836	C/T	Intronic	CTNNBIP1	0	6.2698E-07
T2	1	54569850	Gga_rs317606057	A/C	Intronic	CHST11	0	7.6804E-07
T2	23	1001873	Gga_rs313364203	C/G	Upstream	LOC101751071	1483	1.0809E-06
T2	23	1002885	Gga_rs317686539	A/G	Upstream	LOC101751071	471	1.0809E-06
T2	4	79854217	Gga_rs312463697	C/T	Intronic	SORCS2	0	1.1829E-06
T2	4	81304194	Gga_rs316862146	A/G	Downstream	LOC101751254	27493	1.7514E-06
T2	21	3599980	Gga_rs312697407	C/T	Intron	UBE4B	0	1.9675E-06
T2	12	16146559	Gga_rs15665176	C/T	Upstream	LOC101748541	36147	2.1375E-06
T2	2	105172491	Gga_rs313252901	C/T	Upstream	LOC101749211	81928	3.5318E-06
T2	1	53583341	Gga_rs13653575	C/T	Intronic	CRY1	0	3.8504E-06
T2	1	53605348	Gga_rs317194543	A/G	Intronic	C1H12ORF23	0	3.8504E-06
T2	1	54673895	Gga_rs312937613	C/G	Intronic	CHST11	0	3.9E-06
T3	4	3773061	Gga_rs316089873	C/T	Intronic	GPC4	0	4.2431E-07
T3	4	3849017	Gga_rs312835522	C/T	Intronic	GPC3	0	4.2431E-07
T3	4	3894709	Gga_rs15566236	-/CCTCTA	Intronic	TLN2	0	4.2431E-07
T3	2	98845985	Gga_rs14224061	C/T	Intronic	LOC421049	0	1.9543E-06
T3	2	98799865	Gga_rs14224029	C/T	Upstream	LOC421049	12448	2.7664E-06
T3	2	98779918	Gga_rs314376032	C/T	Downstream	RAB12	2751	3.0104E-06
T3	2	98874042	Gga_rs314310622	C/T	Intronic	LOC421049	0	3.2444E-06
T3	2	98800823	Gga_rs315711449	A/G	Upstream	LOC421049	11490	4.5834E-06
T4	19	2014848	Gga_rs317673228	C/G	Downstream	AUTS2	5428	5.4979E-07
T4	19	2019559	Gga_rs314708256	A/C	Downstream	AUTS2	10139	5.4979E-07
T4	12	845905	Gga_rs317911278	C/G	Intronic	SFMBT1	0	5.73E-07
T4	12	745686	Gga_rs313538765	C/T	Intronic	SPCS1	0	1.2433E-06
T4	26	2576839	Gga_rs313009124	A/T	Intronic	LOC419851	0	3.0497E-06
T4	13	16756745	Gga_rs317892933	A/G	Intronic	ARHGAP26	0	3.8939E-06

**Table 4:** The information of network generated by IPA analysis.

The 10^th^ Top Diseases and Functions	Gene Symbol (n = 17)	Focus Molecule
Developmental disorder; Endocrine system disorders; Hereditary disorder; Organismal injury and abnormalities; Reproductive system disease; Skeletal and muscular disorders; Cellular compromise; Cellular function and maintenance; Protein degradation; Protein synthesis.	TET2, **TET3^1^**, **CHST11^2^**, L-dopa, **GPC3^3^**, **CTNNBIP1^2^**, dopamine, **GPC4^3^**, **SORCS2^1,2^**, PPP2R5E, **CRY1^2^**, BDNF, NGF, NGFR, **HTT^1,2^**, **ARHGAP26^2,4^**, KLF5	17 (9 Molecules associated with aggressive-behaviour from GWAS-SNPs results)

The Gene Symbol in “Bold” indicates that the gene was significantly associated with aggression behaviour traits. “1” indicates that the gene was associated with T1 aggressive-behaviour phenotype; “2” indicates that the gene was associated with T2 aggressive-behaviour phenotype; “3” indicates that the gene was associated with T3 aggressive-behaviour phenotype; “4” indicates that the gene was associated with T4 aggressive-behaviour phenotype.
