# Supplementary material for: Genome-wide association study of aggressive behaviour in chicken
Source: Sci Rep. 2016 Aug 3;6:30981. doi: 10.1038/srep30981 (PMC4971532; doi:10.1038/srep30981)
Supplement: Supplementary Information [file srep30981-s1.pdf]

Genome-wide association study of aggressive behaviour in chicken

Zhenhui Li[1, 2], Ming Zheng[1, 2], Bahareldin Ali Abdalla[1, 2], Zhe Zhang[1, 2], Zhenqiang Xu[1, 2, 3], Qiao Ye[1, 2], Haiping Xu[1, 2], Wei Luo[1, 2], Qinghua Nie[1, 2*], and Xiquan Zhang[1, 2]

[1]Department of Animal Genetics, Breeding and Reproduction, College of Animal Science, South China Agricultural University, Guangzhou 510642, Guangdong, China.

[2]Guangdong Provincial Key Lab of Agro-Animal Genomics and Molecular Breeding and Key Lab of Chicken Genetics, Breeding and Reproduction, Ministry of Agriculture, Guangzhou 510642, Guangdong, China.

[3]Wens Nanfang Poultry Breeding Co., Ltd, Yunfu 527400, Guang dong, China.

*To whom correspondence should be addressed. Tel: 86-20-85285759; Fax: 86-20-85280740; Email: nqinghua@scau.edu.cn.

Figure S1. Quantile-Quantile plot and genome inflation factor lambda describing the deviation between observed and expected –log10 P-values. The red solid diagonal line shows the expected values while the green plots show the observed values. T1: Number of fighting times during the whole recording period (16 days); T2: Number of fighting times in days with frequencies not less than 4 times per day; T3: Number of days for chicken showed fighting; T4: Number of days for chicken showed fighting with frequencies not less than 4 times per day.

T1：Genome Inflation Factor Lambda=1.00000

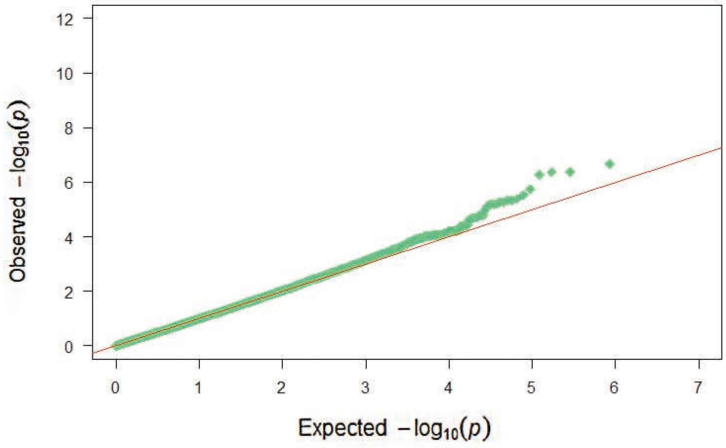

T2：Genome Inflation Factor Lambda=1.00000

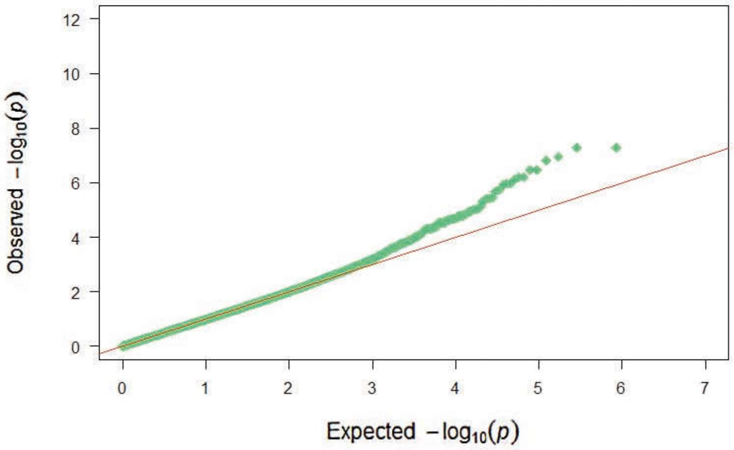

T3：Genome Inflation Factor Lambda=1.00000

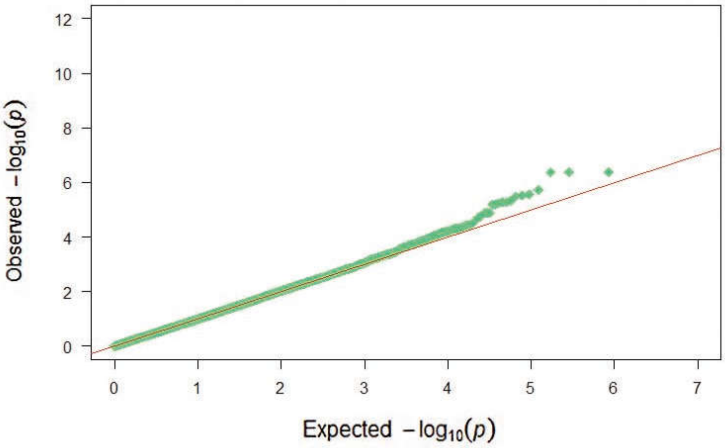

T4：Genome Inflation Factor Lambda=1.00000

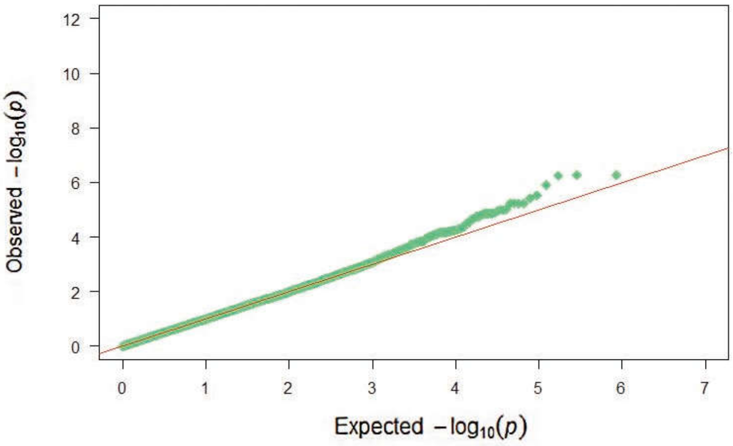

Figure S2. Chicken daily body weight and daily feed intake measurement.

Automatic feeding recording system was used to identify individuals and recording daily body weight and daily feed intake by an infrared (detector) scanning electronic chip (Guangxing Poultry Equipment Group CO., LTD; Guangdong Province, China) which was inserted between two wattles of each male.

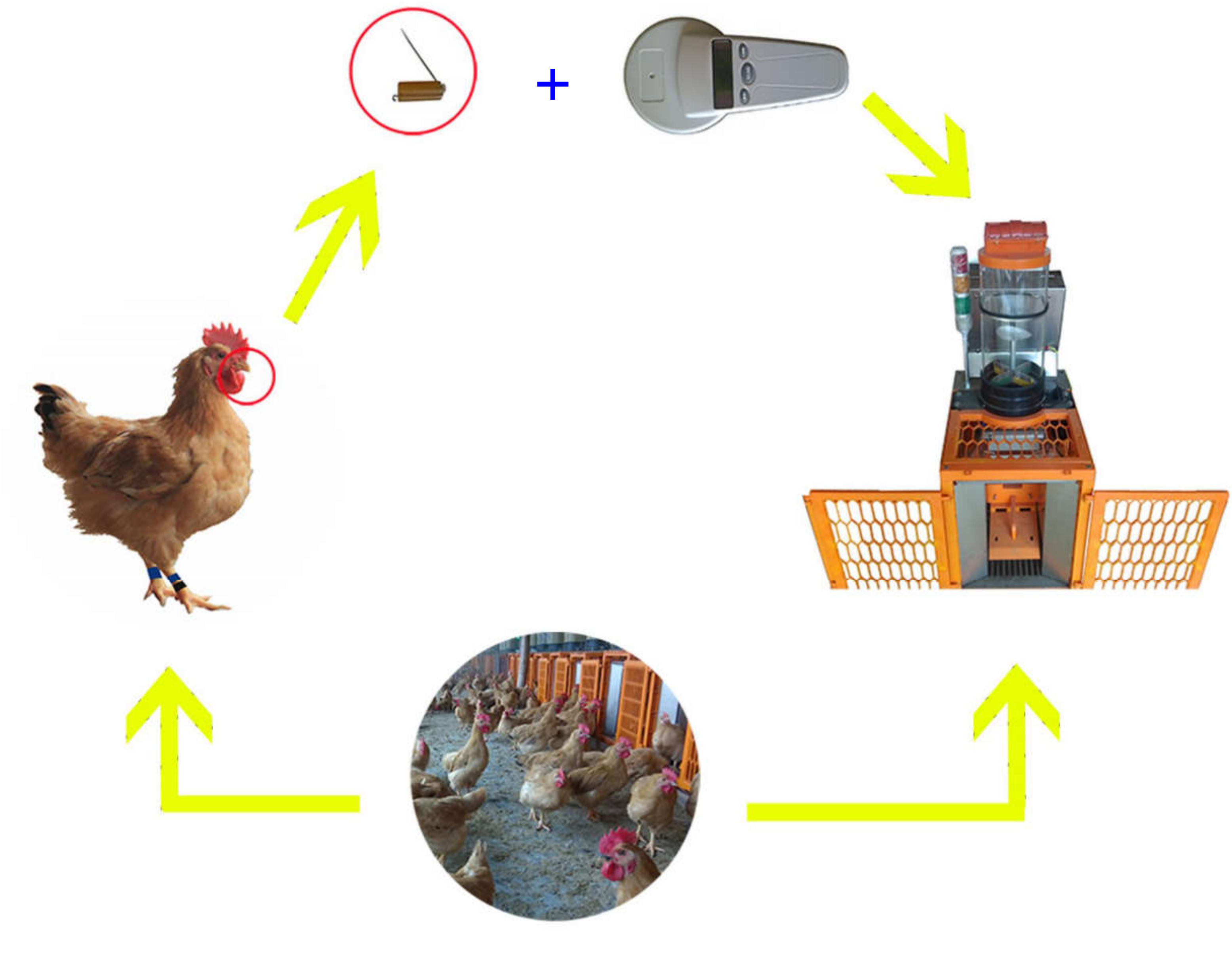

Figure S3. Chicken aggressive behaviour trait measurement.

Behavioural testing of 265 male chickens was begun when the birds were at 60 days of age, in order to record the aggressive behaviour for individuals behaviour, each male was feet-banded with specific color. Seven specific color feet-bandeds, including green, red, yellow, black, white, blue and variegated color, are representing the number from 1 to 7, respectively. For example, the chicken was given blue and black feet-banded in right foot and blue feet-banded in left foot; the chicken ID number is 646 **(a)**. All birds were given 7 days to adapt to the new condition. After the adaptation period, male aggression behaviour was recorded from 67 to 82 days of age, by a team of skilled observers (3 persons), and each of them was responsible for recording the aggression trait after standing on the middle of the one third (5.7 m × 3.0 m) of the male chickens floor pen. Before the male aggressive recording process, the observers waited for 5 to 8 minutes until the chicken's activity was unaffected by the existence of the observers. The definition of aggressive behaviour is based on the ethogram of Väisänen, where no feather pulling was involved. The following behavioural features were recorded in this study: including threats, attacks, chase, aggressive peck, fight and leap. So, the aggressive behaviours were observed two times a day for 16 days; testing took place between 9:00 and 11:00 am, and 03:00 and 05:00 pm **(b)**.

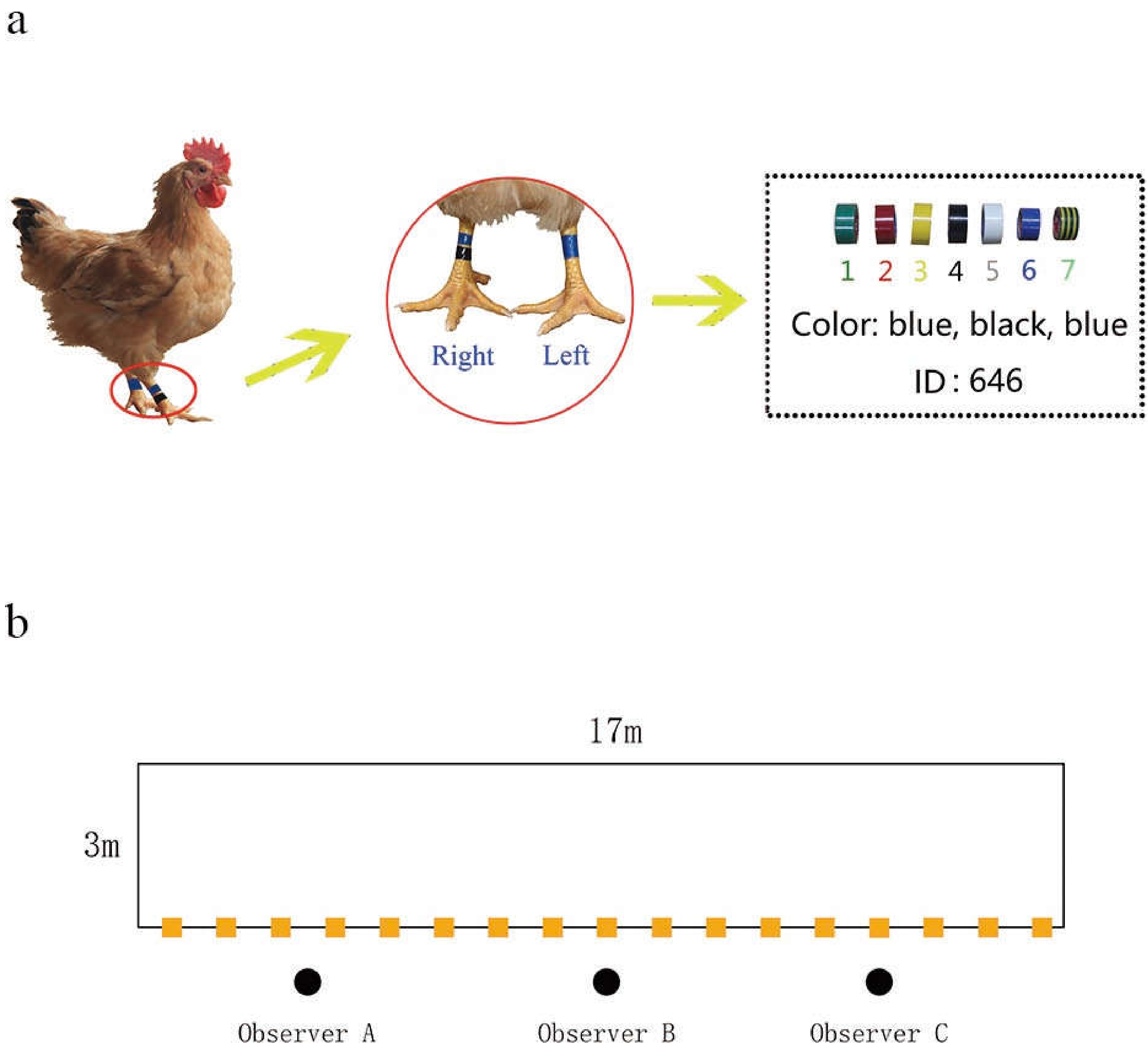
a
Right
Left
1 2 3 4 5 6 7
Color: blue, black, blue
ID : 646
b
17m
3m
Observer A
Observer B
Observer C

Table S1. Distributions for SNPs in 600K Axiom® chicken genotyping array and their conditions after quality control (QC). UN stands for SNPs are assigned to unknown position.

Chr	No. SNP in chip	Average distance (Kb)	No. SNP remained after QC	Average distance after QC (Kb)
1	98565	2.04	84182	2.39
2	62044	2.50	53601	2.89
3	55166	2.06	47808	2.38
4	41737	2.26	36395	2.59
5	29550	2.11	25673	2.42
6	21192	1.76	18403	2.03
7	20870	1.84	18064	2.12
8	16771	1.83	14197	2.16
9	17519	1.46	15027	1.70
10	18337	1.23	15080	1.50
11	13490	1.63	11212	1.96
12	14321	1.43	11810	1.74
13	10915	1.73	9473	2.00
14	12768	1.24	10591	1.49
15	10170	1.28	8524	1.52
16	536	0.81	329	1.32
17	9089	1.23	7381	1.52
18	9367	1.17	8015	1.36
19	8797	1.13	7365	1.35
20	9332	1.50	7729	1.81
21	8654	0.81	7112	0.98
22	4522	0.87	3498	1.13
23	6465	0.93	5270	1.15
24	7492	0.85	5831	1.10
25	2390	0.85	1835	1.11
26	6113	0.83	4925	1.04
27	5511	0.88	4274	1.13

28	5338	0.85	4162	1.08
LGE22	205	4.40	150	6
LGE64	74	0.18	51	0.33
Z	25081	2.97	13818	5.40
W	14	18.54	0	-
UN	7503	-	6235	-
Total	559898	1.84	468020	2.21

Table S2. Block information. UN stands for SNPs are assigned to unknown position.

Chr	Total number of SNPs	Total number of blocks	Total number of interblocks SNPs	Total number of independent test
1	84182	13567	23433	37000
2	53601	8826	16333	25159
3	47808	8032	14481	22513
4	36395	5979	10319	16298
5	25673	4225	7652	11877
6	18403	3110	5979	9089
7	18064	3007	5673	8680
8	14197	2098	4165	6263
9	15027	2466	4096	6562
10	15080	2426	4399	6825
11	11212	1866	3673	5539
12	11810	1777	3593	5370
13	9473	1670	3382	5052
14	10591	1626	3046	4672
15	8524	1344	2481	3825
16	329	42	181	223
17	7381	1190	2652	3842
18	8015	1436	2935	4371
19	7365	1341	2644	3985
20	7729	1312	2313	3625
21	7112	1195	2374	3569
22	3498	568	1393	1961
23	5270	944	2030	2974
24	5831	891	1815	2706
25	1835	317	650	967
26	4925	907	1786	2693
27	4274	682	1680	2362
28	4162	688	1631	2319
LGE22	150	27	75	102
LGE64	51	0	51	51

Z	13818	782	1390	2172
W	0	0	0	0
UN	6235	954	4188	5142
Total	468020	75295	142493	217788

Table S3. Primer information for qPCR.

Target gene	Primer sequence (5’ to 3’)	Products (bp)
SORCS2	F : GGCATTTACTACTCCATCCT<br>R : AACTTTCCCATCAATCTTCT	135
NGF	F: AGAGTAACGGACAGCACATTG<br>R: GAACAGGACCCGAGAAGACC	149
HTT	F: ACTTCTTTAGGTGGCATTG<br>R: TGACATCTGATCGGGTCT	151
NGFR	F: CCCTGTGAACCAGACGCCTTCC<br>R: TCCTCCTTGTAGCCCAACTCCC	249
L-dopa	F: TTTTGCCTACTTCCCGTCAG<br>R: CTTGCCCATCCCTTCCAG	191
DRD1	F: AGTGACACTTTCAGGGAACAGCA<br>R: GCCGCCTCTTCTCCTCATTTA	101
DRD2	F: CCTCCTCATCTTTGTCATTGTG<br>R: CCGACTGAACCTCCACTCCC	187
DRD3	F: CTTTGGCTTCAATACTACAGG<br>R: TTGTCTTAGCACGAGGTAAAT	145
DRD4	F: TCTGCGATGCCCTGATGACC<br>R: TGGGACTGAAACAGCGATAAAC	101
DRD5	F: GGGGCTTTCTGCAACGTCTG<br>R: GCCGCTGGGTCATCTTCCTC	139
β-actin	F: GATATTGCTGCGCTCGTTG<br>R: TTCAGGGTCAGGATACCTCTTT	194

Ingenuity Pathway Analysis (IPA)

IPA is a web-based software from Ingenuity Systems®, (QIAGEN Redwood City, CA, USA) provides a comprehensive database of known networks and pathways that are continuously being updated based on published works on gene functions and interactions, which enables investigators to analyze data derived from RNA sequencing, small RNA sequencing, and microarray experiments including SNP, miRNA, proteomics, metabolomics, and even small-scale trials (e.g., qPCR) that generate gene or chemical lists. It also provides search for targeted information on genes, proteins, diseases, drugs and chemicals, as well as building interactive models of experimental systems. In addition, IPA uses computational algorithms to identify local networks that are particularly enriched for the focus genes with a score given. The network score is a numerical value used to rank networks which is based on the hyper-geometric distribution, is calculated with the right-tailed Fisher's Exact Test, and is represented as a negative log of this *P* value. Moreover, IPA's data analysis enables understanding the significance of data or targets or candidate biomarkers in relation to larger biological or chemical systems. Additional information can be found on IPA website as following: www.ingenuity.com.
